# Postoperative Exercise Rehabilitation and Postural Outcomes Among Transgender Individuals Undergoing Chest Masculinization Surgery

**DOI:** 10.7759/cureus.91840

**Published:** 2025-09-08

**Authors:** Michael Edgar, Sam H Jiang, Rachael Johnson, Amanda Vevers, Norah Oles, Matthew Ranzer, Julia Corcoran

**Affiliations:** 1 Plastic Surgery, University of Illinois, Chicago, USA; 2 Neurosurgery, University of Illinois, Chicago, USA; 3 Psychiatry, University of Illinois, Chicago, USA

**Keywords:** chest masculinization surgery, exercise, gender-affirming surgery, posture, transgender health

## Abstract

Purpose

This pilot study evaluated the feasibility and initial effectiveness of a structured postoperative exercise rehabilitation program on postural outcomes for transgender individuals undergoing chest masculinization surgery (CMS).

Methods

A prospective cohort study was conducted at University of Illinois (UI) Health (Chicago, IL) from January to December 2023. Thirty-four participants (aged 18-25) were enrolled and divided into a rehabilitation group (n=20) and a standard care group (n=14). The rehabilitation group followed an eight-week exercise protocol targeting postural improvements, while the standard care group received no formal rehabilitation. Postural metrics, including wall-to-tragus and scapular tip distance, were measured preoperatively and at multiple postoperative intervals up to three months. Statistical analyses included Mann-Whitney U tests for continuous variables and Pearson’s χ² tests for categorical variables.

Results

Adherence to the rehabilitation program was high, with eight participants (40%) reporting strong compliance. The rehabilitation group demonstrated a significant improvement in scapular tip distance (-2.69 cm, p=0.003), suggesting enhanced scapular retraction. Other postural metrics showed trends toward improvement but did not reach statistical significance. Qualitative feedback indicated high satisfaction with the rehabilitation program.

Conclusion

This study supports the feasibility and acceptability of a postoperative rehabilitation program for transgender individuals undergoing CMS. While improvements in posture were observed, larger studies with extended follow-up are needed to confirm these findings. Future research should also explore potential psychosocial benefits of postoperative rehabilitation in this population.

## Introduction

Gender dysphoria is characterized by feelings of distress due to lack of congruency between between one’s sex assigned at birth and self-identified gender [1]. The World Professional Association of Transgender Health Standards of Care (WPATH), version eight, currently offers interdisciplinary guidelines for treating individuals with gender dysphoria [2,3]. Treatment options for gender-affirming care include, but are not limited to, hormone replacement therapy (HRT), psychotherapy, and surgery [2]. Gender-affirming surgery (GAS) is typically described as any surgery that provides the individual with improved concordance between their physical appearance and gender identity [2].

The rate of individuals undergoing GAS has increased in recent years along with favorable changes in the sociopolitical landscape and insurance coverage [2]. Chest masculinization surgery, the focus of this study, is a typical part of the transition process for transgender males. Thus, the descriptor "transmasculine" will be employed when referring to this patient population. However, it is crucial to recognize that chest masculinization can be a part of the transition process for any individual for whom breast tissue is a source of dysphoria, including individuals who may not identify with the descriptor of “masculine.”

Chest masculinization can be achieved via a variety of techniques depending on the goals of the patient as well as physical factors such as breast volume, degree of excess skin, nipple-alveolar complex (NAC) size and position, and skin elasticity [2]. As with all surgery, there is risk to undergoing GAS; pooled rates of postoperative complication of chest masculinization have been reported to be as high as 10%, with hematoma being the most common complication [2]. Less common risks associated with chest masculinization surgery were observed to be loss of the NAC and reoperation requirements [2]. Despite significant complication rates, patients still consistently reported high satisfaction rates.

Despite the increased prevalence and normalization of chest masculinization, the area of postoperative rehabilitation in this patient population remains sparse particularly in relation to the presence of hyperkyphosis in preoperative transmasculine patients. A postural presentation often observed for transmasculine patients’ preoperative posture is hyperkyphosis, as individuals round their shoulders and posture forward as a means of minimizing and disguising their breast tissue. The age of onset of this posture varies, but it anecdotally develops when transmasculine children undergo estrogen-directed pubertal changes that cause growth of breast tissue. As gender-affirming surgery of any type is rarely offered to minors, this posture can persist for years and become difficult to correct even after removal of breast tissue. In addition to the psychological aspects associated with this, hyperkyphosis of the thoracic spine has also been associated with chronic pain [4].

Postoperative rehabilitation is heavily described in other fields of surgery, such as breast augmentation after mastectomy in patients with cancer [5]. Breast cancer surgery postoperative exercise rehabilitation has shown a variety of positive effects related to functional outcomes of shoulder mobility, improved strength, wound healing, and psychological perceptions of recovery [5,6]. Exercise rehabilitation has also been shown to improve age-related hyperkyphotic posture [6,7].

The applicability of rehabilitation literature following breast reconstruction after oncologic procedures is limited, given the substantial difference between gender-affirming chest masculinization and breast cancer surgery, particularly regarding pectoral fascia damage, lymph node removal, and tissue irradiation. Given the currently limited information related to the utility of postoperative exercise rehabilitation for patients undergoing chest masculinization GAS, we designed a study to determine if a prescribed exercise program may improve different metrics of recovery. Our primary objective was to evaluate within-subject change in quantitative postural metrics from preoperative baselines to postoperative follow-ups. Secondary objectives were feasibility and acceptability of the program (adherence, satisfaction), and exploratory subgroup analyses.

## Materials and methods

Ethical approval and informed consent

This study was conducted in accordance with the ethical principles outlined in the Declaration of Helsinki (2013 revision) and was approved by the Institutional Review Board (IRB) of University of Illinois (UI) Health, Chicago, IL (IRB Protocol Number: STUDY2022-1273). Written informed consent was obtained from all participants prior to study enrollment. Participants were provided with detailed information regarding study procedures, risks, and benefits, and they were given the opportunity to ask questions before consenting.

Study design

In this prospective cohort study conducted at the Primary and Specialty Care Gender Affirming Surgery Clinic, UI Health, in Chicago, IL, participants were recruited sequentially from patients undergoing routine care at the Gender Affirming Surgery Clinic who were scheduled to undergo chest masculinization surgery. Inclusion criteria consisted of individuals aged 18-25 years, meeting the World Professional Association for Transgender Health (WPATH) Standards of Care, version eight (SOC v8) criteria for gender dysphoria, and who had no prior chest surgery [3]. Exclusion criteria included minors, patients with prior chest surgery, spinal pathology that impeded postural improvements (i.e. pectus, scoliosis), return to surgery, wound healing issues after day 14 post-operation, and pre-existing conditions that prevented the ability to perform exercise rehabilitation exercises. A follow-up at two to three months was required to determine if the changes were sustainable and not a temporary effect of the exercise program.

**Figure 1:**
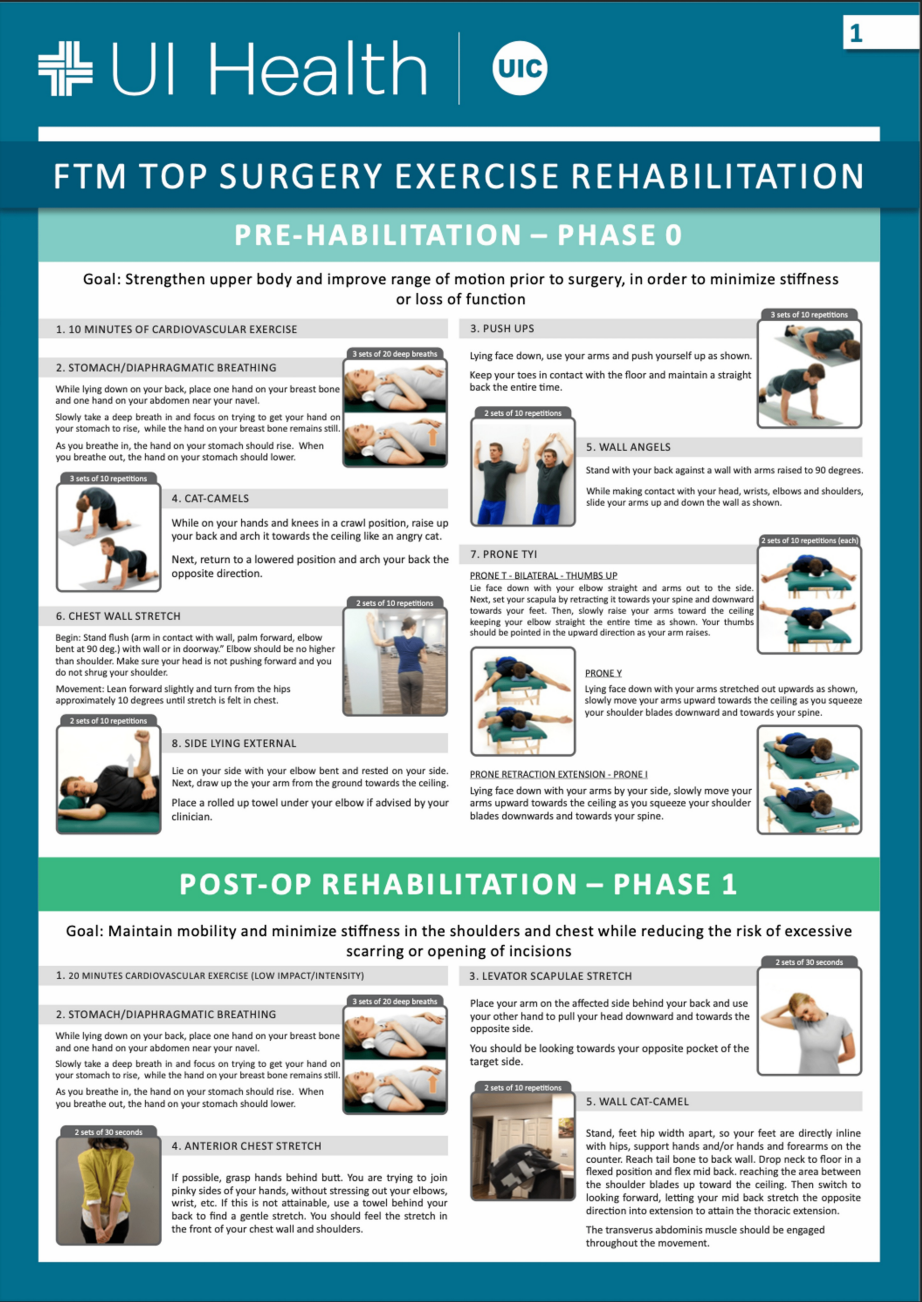
Post-Operative Exercise Rehabilitation Program Page One Image credit: Created by the authors as part of the postoperative rehabilitation materials developed at the University of Illinois (UI) Health. These images are original and have not been reproduced from any external source. Permission for use has been granted by the author (Michael Edgar), who holds the intellectual property rights. All individuals depicted are volunteers and not patients.

**Figure 2:**
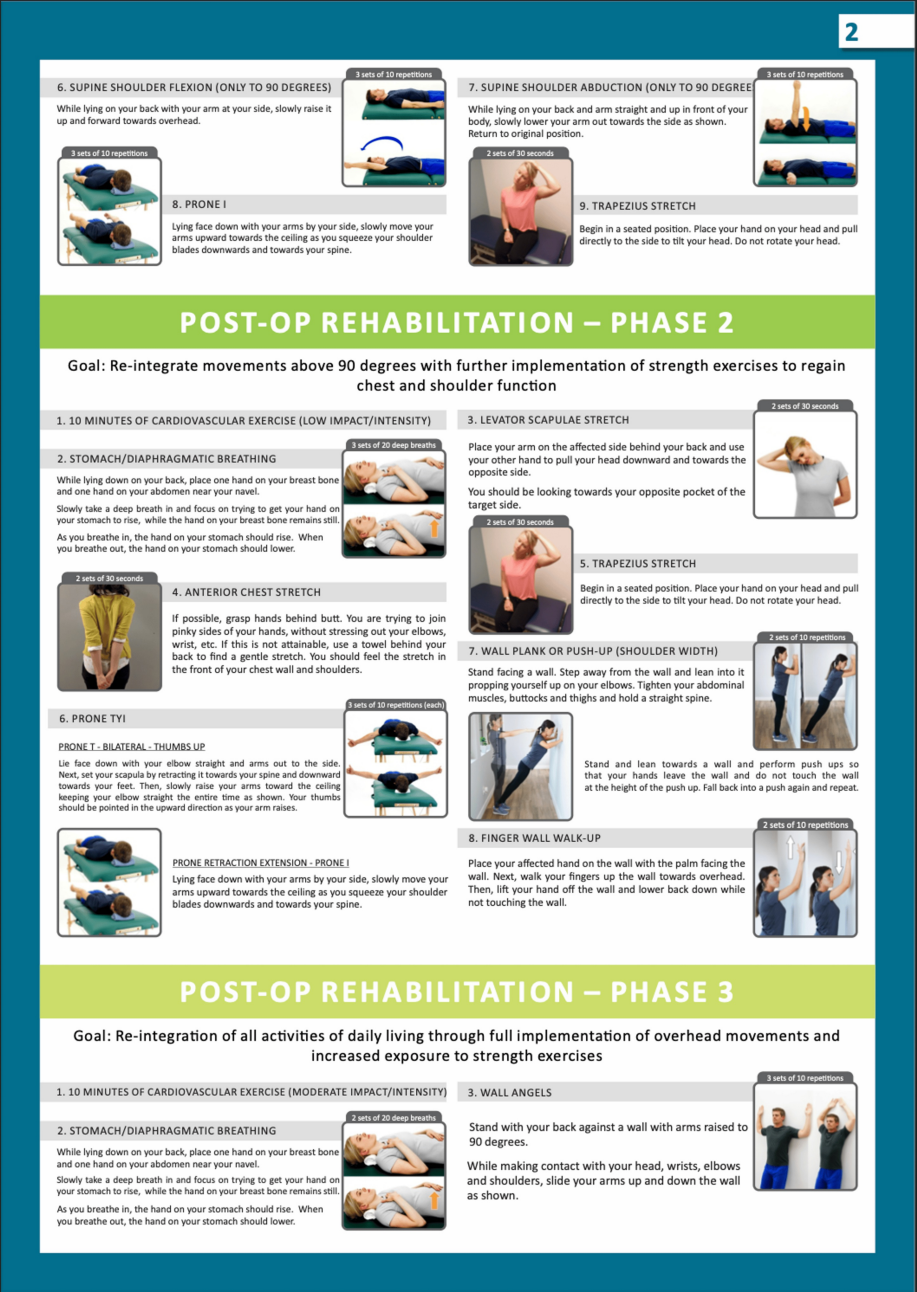
Post-Operative Exercise Rehabilitation Program Page Two Image credit: Created by the authors as part of the postoperative rehabilitation materials developed at the the University of Illinois (UI) Health. These images are original and have not been reproduced from any external source. Permission for use has been granted by the author (Michael Edgar), who holds the intellectual property rights. All individuals depicted are volunteers and not patients.

**Figure 3:**
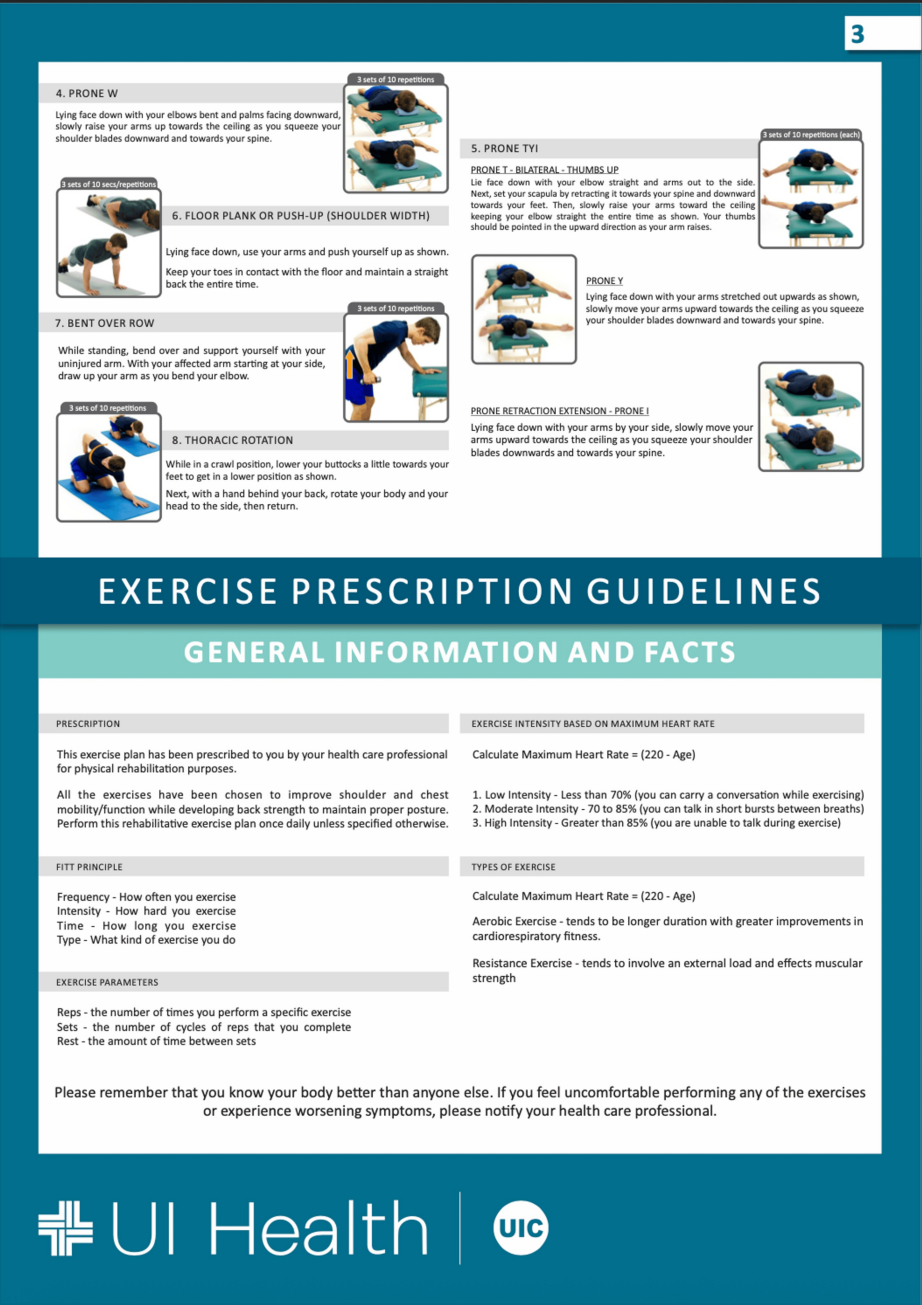
Post-Operative Exercise Rehabilitation Program Page Three Image credit: Created by the authors as part of the postoperative rehabilitation materials developed at the University of Illinois (UI) Health. These images are original and have not been reproduced from any external source. Permission for use has been granted by the author (Michael Edgar), who holds the intellectual property rights. All individuals depicted are volunteers and not patients.

Eligible participants were approached during their clinic visits by a designated study personnel to discuss the purpose of the study, explain the exercise program and obtain informed consent. Patients were assigned to either the rehabilitation group or the standard-of-care group, which did not receive a formal rehabilitation protocol, based on patient preference to undergo the rehabilitation program or not. The rehabilitation intervention comprised an eight-week structured postoperative exercise rehabilitation program, detailed in Figures 1-3.

Data collection

Objective Measures

**Figure 4:**
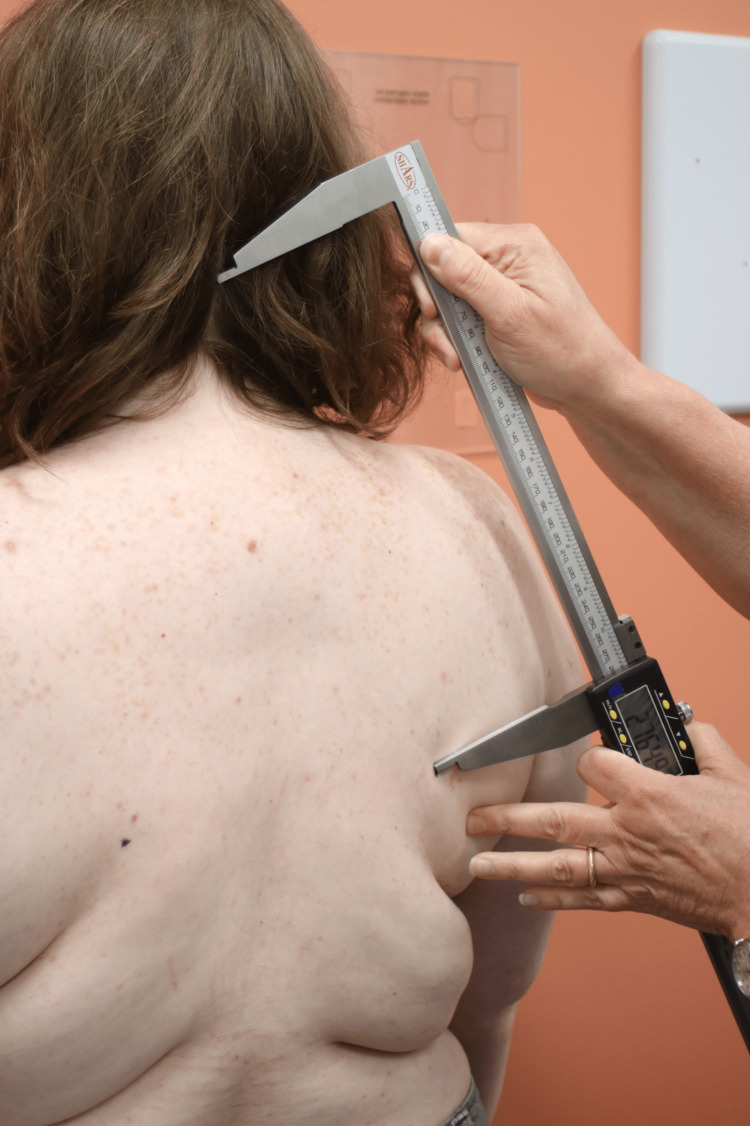
Measuring External Occipital Protuberance to Scapular Tip Distance

**Figure 5:**
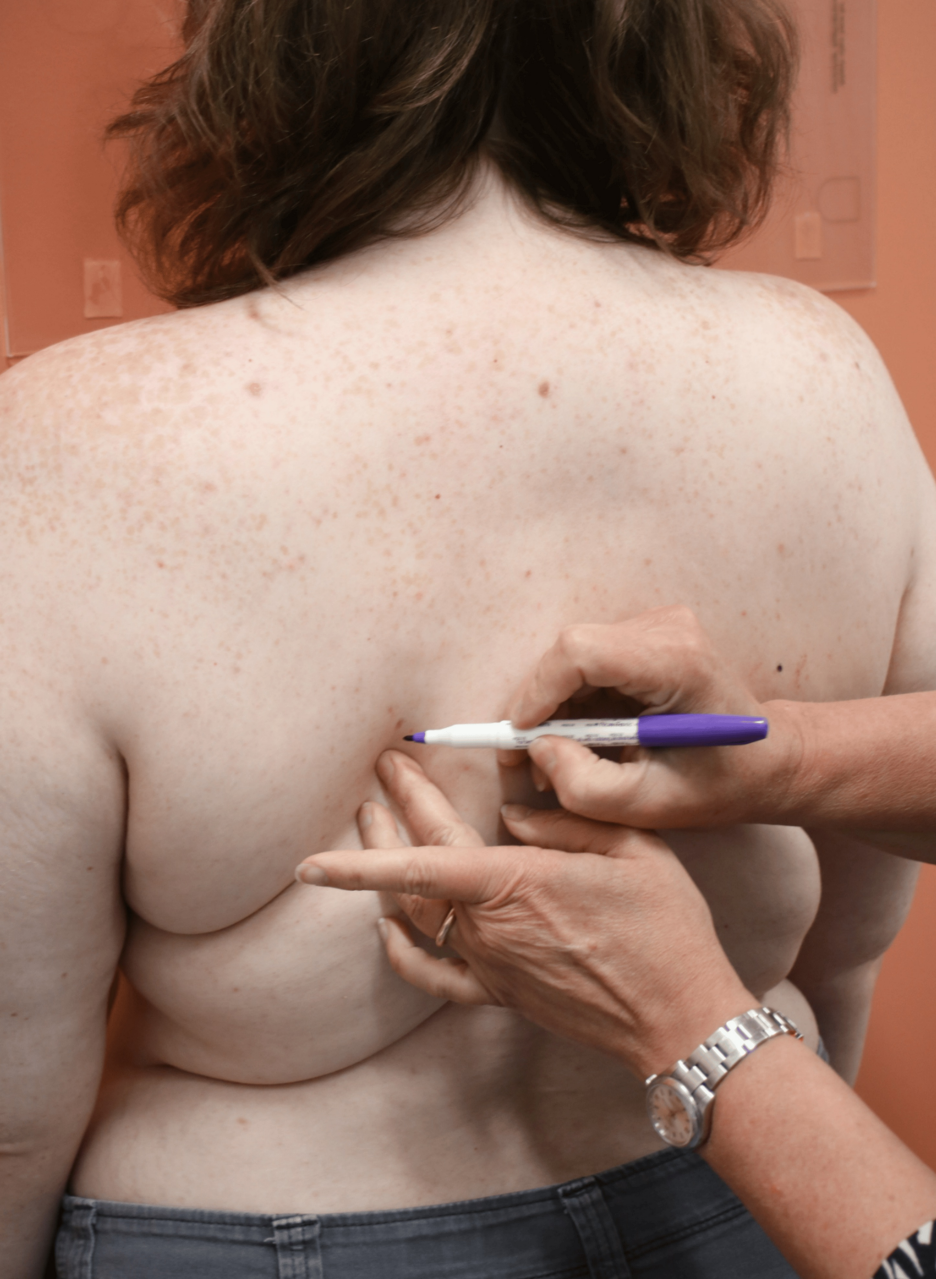
Landmarking Scapular Tips

**Figure 6:**
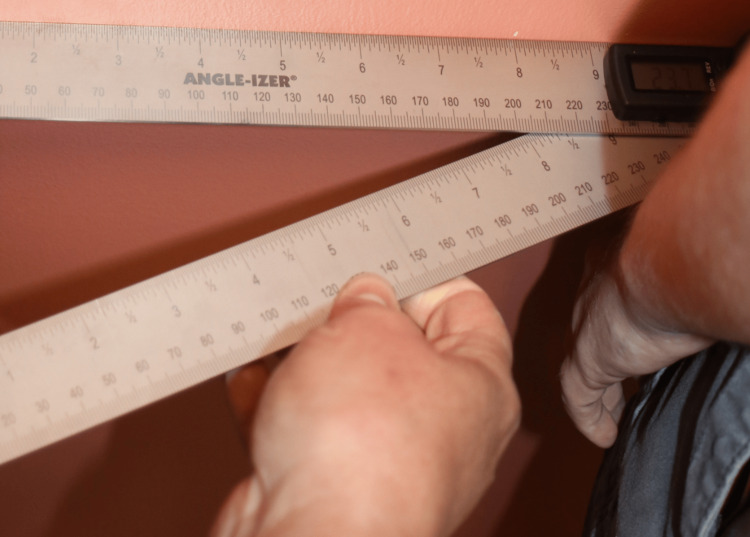
Measuring Degree of Internal Rotation

**Figure 7:**
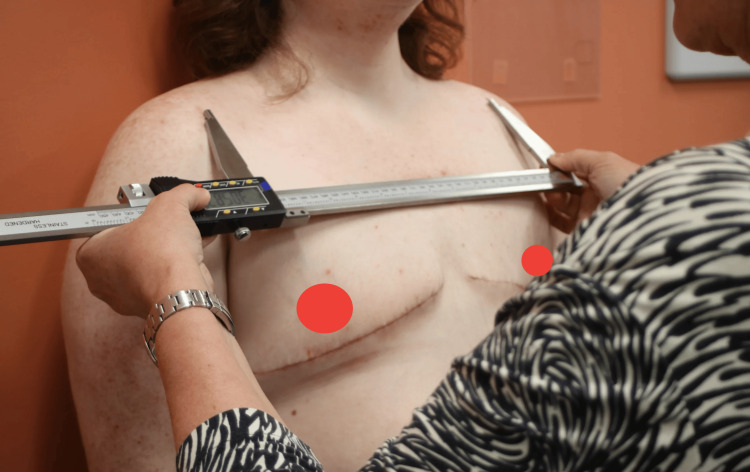
Measuring Acromioclavicular Distance

**Figure 8:**
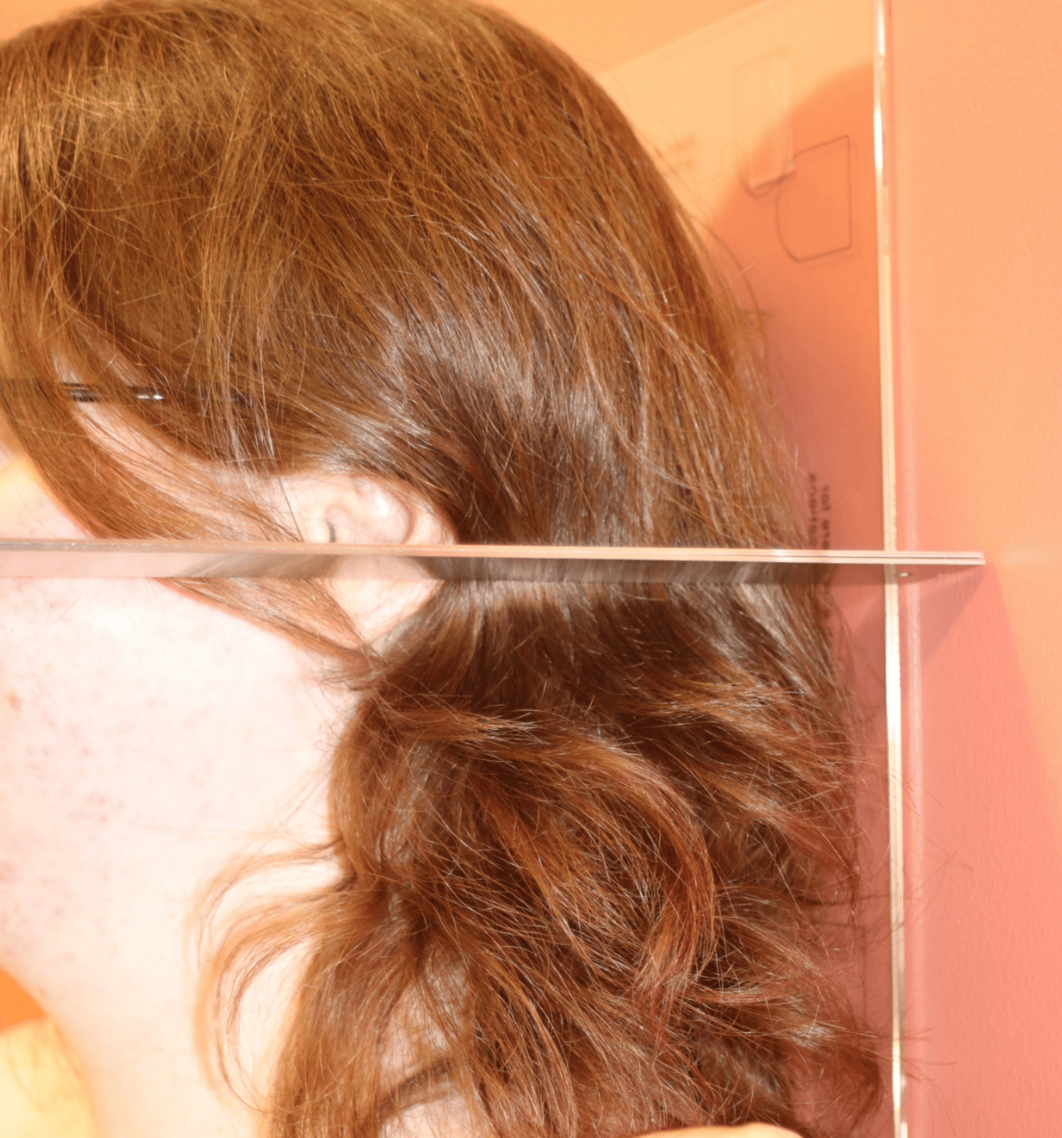
Image 5. Measuring Wall to Tragus Distance

Data was collected at three key time points from January to December 2023: preoperative, two to four weeks postoperative and eight to 12 weeks postoperative. Data collection included both quantitative and qualitative measures. Postural metrics for controls were recorded as part of standard clinical practice. Quantitative measures included wall-to-tragus distance, digital caliper measures for anatomical landmarks and goniometric measurements (Figures 4-8). The wall-to-tragus distance was measured bilaterally from the wall to the tragus, the small, pointed eminence of the external ear (Figure 8). Participants stood with their heels, buttocks, and shoulders touching the wall, and the distance was measured using a digital caliper from the wall to the tragus of both the left and right ears. 

The acromioclavicular (AC) joint distance was another key measurement, taken using a digital caliper to assess the distance between the AC joints (Figure 7). The scapular tip distance was measured from the inferior angle of both scapulae (Figure 5). The external occipital protuberance (EOP) to scapular tip distance was measured bilaterally while seated, providing a metric for assessing postural changes in the upper back and shoulder region (Figure 4). Lastly, shoulder internal rotation was measured using a goniometer with the patient facing the wall (Figure 6). These standardized measurements ensured reliable data collection and allowed for precise tracking of postural changes over time. All quantitative measures were performed by two healthcare providers that were trained in a standardized fashion. 

Subjective Measures

Subjective outcomes were assessed using a patient-reported satisfaction questionnaire at each follow-up visit. Ease of use was evaluated by asking participants to rate the clarity and practicality of the exercise program on a five-point Likert scale, with one indicating "very difficult" and five indicating "very easy." Confidence in correctly performing the exercises was similarly assessed using a five-point Likert scale, ranging from one ("not confident at all") to five ("very confident"). Motivation was measured by self-reported willingness to continue the program after the study, rated on the same five-point scale. Finally, overall satisfaction with the exercise program was assessed, with participants rating their satisfaction on a five-point Likert scale, where one represented "very dissatisfied" and five represented "very satisfied." The questionnaire was aimed at feasibility endpoints and not posture-specific outcomes.

Data analysis

Missing data was imputed using a missing forest approach. Descriptive statistics was performed using Mann-Whitney U tests for continuous variables and Pearson’s χ^2^ tests for binary variables to compare differences between the rehabilitation and standard-of-care groups. Spearman's rank correlation tests were used to evaluate the relationship between total resection mass and postoperative postural changes.

Subgroup analyses were subsequently performed to compare postural changes in left- versus right-handed patients, as well as postural changes based on high, medium, and low self-reported adherence to the exercise protocol within the rehabilitation group. For the latter, one-way analysis of variance was used for continuous variables and Pearson’s χ^2^ tests were used for binary variables. Alpha was set to 0.05 for all analyses. Data processing and statistical analyses were performed in Python 3 (Python Software Foundation, Beaverton, OR, USA) using the PsmPy, SciPy, and statsmodels libraries.

## Results

A total of 34 participants were included in the study. Of those, 20 participants agreed to participate in the exercise rehabilitation program while 14 patients opted for standard-of-care treatment. At baseline, patients in the rehabilitation group were lighter (74.81 lb vs. 94.81 lb, p=0.04) and had lower BMI (27.51 kg/m^2^ vs. 34.55 kg/m^2^, p=0.03) compared to the standard of care group. Otherwise, they did not differ significantly with respect to age, self-identified gender, race/ethnicity, handedness, or living situation (Table 1). In addition, the groups did not differ at baseline in procedure type or medical comorbidities (Table 2). Within the rehabilitation group, 40% reported high adherence to the program, 50% reported medium adherence, and 10% reported low adherence. The self-reported ratings for program satisfaction and likelihood to continue, ranging from 1 to 5 with 5 being the highest, were 4.25 and 4.05, respectively (Table 3).

**Table 1:** Baseline patient characteristics. P-values for continuous variables were calculated using Mann-Whitney U tests and are accompanied by their corresponding U-values. P-values for categorical variables were calculated using Pearson’s chi-square (χ²) tests with corresponding χ² values listed. *P ≤ 0.05 indicates statistical significance. BMI = Body Mass Index; SD = Standard Deviation. * = significant

Variable	All (n=34)	Rehab (n=20)	No Rehab (n=14)	p-Value (χ² test)	p-Value (Mann-Whitney test)
Age (years), mean±SD	20.74±2.39	20.65±2.43	20.86±2.41		0.81
Gender					
Male	26 (76.47)	14 (70.00)	12 (85.71)	0.42	
Non-Binary	8 (23.53)	6 (30.00)	2 (14.29)	0.42	
Race/Ethnicity					
White	26 (76.47)	17 (85.00)	9 (64.29)	0.23	
Black	4 (11.76)	1 (5.00)	3 (21.43)	0.28	
Hispanic	1 (2.94)	0 (0.00)	1 (7.14)	0.41	
Left-Handed	9 (26.47)	4 (20.00)	5 (35.71)	0.44	
Right-Handed	25 (73.53)	16 (80.00)	9 (64.29)	0.44	
Lives with Family	24 (70.59)	14 (70.00)	10 (71.43)	0.99	
Occupation					
Corporate	1 (2.94)	1 (5.00)	0 (0.00)	0.99	
Food Service	4 (11.76)	3 (15.00)	1 (7.14)	0.63	
Healthcare	1 (2.94)	1 (5.00)	0 (0.00)	0.99	
Other	2 (5.88)	2 (10.00)	0 (0.00)	0.5	
Student	26 (76.47)	13 (65.00)	13 (92.86)	0.1	
Height (m), mean±SD	1.65±0.06	1.65±0.06	1.65±0.05		0.86
Weight (kg), mean±SD	83.04±28.22	74.81±19.67	94.81±34.68		0.04*
BMI (kg/m^2^), mean±SD	30.41±9.41	27.51±6.70	34.55±11.30		0.03*

**Table 2:** Baseline procedural characteristics. P-values for continuous variables were calculated using Mann-Whitney U tests and are accompanied by their corresponding U-values. P-values for categorical variables were calculated using Pearson’s chi-square (χ²) tests with corresponding χ² values listed. P≤0.05. ADHD: Attention deficit hyperactivity disorder; ASD: adult spinal deformity; EBL: estimated blood loss; OCD: obsessive compulsive disorder; SD: standard deviation

Variable	All (n=34)	Rehab (n=20)	No Rehab (n=14)	p-Value (χ² test)	p-Value (Mann-Whitney test)
Procedure Type					
Double Incision	31 (91.18)	18 (90.00)	13 (92.86)	0.77	
Peri-Areolar	3 (8.82)	2 (10.00)	1 (7.14)	0.77	
Nipple Sparing	32 (94.12)	20 (100.00)	12 (85.71)	0.081	
Operative Duration (min), mean ± SD	176.68 ± 43.55	186.91 ± 42.82	162.06 ± 41.75		0.073
EBL (mL), mean ± SD	40.10±18.11	42.12±21.88	37.21±10.83		0.94
Medical History					
ADHD	1 (2.94)	0 (0.00)	1 (7.14)	0.23	
Anxiety	21 (61.76)	11 (55.00)	10 (71.43)	0.33	
ASD	1 (2.94)	0 (0.00)	1 (7.14)	0.23	
Asthma	1 (2.94)	1 (5.00)	0 (0.00)	0.4	
Bipolar Disorder	2 (5.88)	1 (5.00)	1 (7.14)	0.79	
Bulimia Nervosa	1 (2.94)	1 (5.00)	0 (0.00)	0.4	
Depression	16 (47.06)	9 (45.00)	7 (50.00)	0.77	
Diabetes	1 (2.94)	1 (5.00)	0 (0.00)	0.4	
OCD	1 (2.94)	0 (0.00)	1 (7.14)	0.23	
Pectus Deformity	2 (5.88)	2 (10.00)	0 (0.00)	0.22	
Psoriasis	1 (2.94)	0 (0.00)	1 (7.14)	0.23	

**Table 3:** Exercise Rehabilitation Program Adherence and Satisfaction Among Participants in the Rehab Group SD, standard deviation. This table includes only participants who enrolled in the rehabilitation group (n=20). Adherence to the exercise program was self-reported using a five-point Likert scale and assessed at follow-up visits and via phone check-ins. High adherence = performing the program 6–7 days/week. Medium adherence = 4–5 days/week. Low adherence = 1–3 days/week The program was completed unsupervised at home.

Variable	Value
Adherence to Program	
High	8 (40.00)
Medium	10 (50.00)
Low	2 (10.00)
Program Satisfaction (1-5), mean±SD	4.25±0.91
Likelihood to Continue Program (1-5), mean±SD	4.05±0.83

Comparing pre-to-postoperative changes in posture, there were no significant differences between the rehabilitation and standard of care groups (Table 4), based on handedness (Table 5), or on subgroup analysis on the rehabilitation group based on program adherence (Table 6). Table 7 presents the mean values and standard deviations of postural measurements across all participants at the preoperative visit, first postoperative visit (two to four weeks), and second postoperative visit (eight to 12 weeks). Of these, only scapular tip distance showed a statistically significant change across timepoints (pre-op to second post-op -2.69 cm, p=0.003). There was no difference in living situation and exercise rehabilitation program adherence (Table 8).

**Table 4:** Pre-to-postoperative changes in posture between rehabilitation and standard-of-care groups. P-values for continuous variables were calculated using Mann-Whitney U tests and are accompanied by their corresponding U-values. P≤0.05. AC: acromioclavicular; EOP: external occipital protuberance; SD: standard deviation. Postural changes were measured between the first postoperative visit (two to four weeks post-op) and the second postoperative visit (eight to 12 weeks post-op), corresponding to a period of approximately six weeks between visits.

Variable	Postop Visit Number	All (n=34)	Rehab (n=20)	No Rehab (n=14)	p-Value
Wall to Left Tragus (cm), mean±SD	First	-1.30±2.17	-0.87±1.84	-1.91±2.52	0.24
	Second	-2.09±2.96	-1.84±3.30	-2.45±2.46	0.52
Wall to Right Tragus (cm), mean±SD	First	-1.09±2.23	-0.88±2.09	-1.38±2.47	0.65
	Second	-2.14±3.28	-1.94±3.65	-2.42±2.78	0.46
AC Joint (cm), mean±SD	First	-0.69±4.52	-0.47±5.29	-0.99±3.30	0.96
	Second	-2.22±5.04	-2.13±5.85	-2.34±3.81	0.85
Scapular Tip (cm), mean±SD	First	-3.02±3.54	-3.18±3.80	-2.79±3.27	0.82
	Second	-4.10±4.05	-4.28±4.30	-3.86±3.82	0.99
EOP to Left Scapula (cm), mean±SD	First	-2.39±4.10	-2.39±4.30	-2.38±3.97	0.88
	Second	-4.32±4.38	-4.68±4.88	-3.80±3.67	0.71
EOP to Right Scapula (cm), mean±SD	First	-2.45±3.69	-2.68±3.53	-2.13±4.03	0.99
	Second	-4.77±4.63	-5.77±4.87	-3.33±3.99	0.26
Left Internal Rotation (degrees), mean±SD	First	-5.17±10.16	-3.92±8.19	-6.95±12.57	0.5
	Second	-9.38±9.56	-8.92±8.91	-10.04±10.74	0.7
Right Internal Rotation (degrees), mean±SD	First	-4.69±11.63	-2.30±9.20	-8.10±14.09	0.48
	Second	-8.03±11.76	-5.91±11.45	-11.06±11.95	0.7

**Table 5:** Pre-to-postoperative changes in posture based on handedness. P-values for continuous variables were calculated using Mann-Whitney U tests and are accompanied by their corresponding U-values. P≤0.05. AC: acromioclavicular; EOP: external occipital protuberance; SD: standard deviation. Postural changes were measured between the first postoperative visit (two to four weeks post-op) and the second postoperative visit (eight to 12 weeks post-op), corresponding to a period of approximately six weeks between visits.

Variable	Postop Visit Number	All (n=34)	Right-Handed (n=25)	Left-Handed (n=9)	p-Value
Wall to Left Tragus (cm), mean±SD	First	-1.30±2.17	-1.24±2.42	-1.46±1.40	0.76
	Second	-2.09 ± 2.96	-2.05±2.88	-2.21±3.33	0.76
Wall to Right Tragus (cm), mean ± SD	First	-1.09 ± 2.23	-0.99±2.40	-1.35±1.78	0.49
	Second	-2.14 ± 3.28	-2.24±3.37	-1.85±3.21	0.92
AC Joint (cm), mean ± SD	First	-0.69 ± 4.52	0.05± 4.89	-2.73±2.53	0.15
	Second	-2.22 ± 5.04	-1.42±5.32	-4.42±3.51	0.073
Scapular Tip (cm), mean ± SD	First	-3.02 ± 3.54	-2.55±3.43	-4.34±3.71	0.2
	Second	-4.10 ± 4.05	-3.91±4.27	-4.64±3.56	0.53
EOP to Left Scapula (cm), mean ± SD	First	-2.39 ± 4.10	-1.83±4.13	-3.93±3.83	0.1
	Second	-4.32 ± 4.38	-4.18±4.62	-4.70±3.87	0.73
EOP to Right Scapula (cm), mean ± SD	First	-2.45 ± 3.69	-1.93±3.73	-3.90±3.35	0.23
	Second	-4.77 ± 4.63	-4.59±4.75	-5.26±4.48	0.56
Left Internal Rotation (degrees), mean ± SD	First	-5.17 ± 10.16	-3.40±10.67	-10.08±6.86	0.12
	Second	-9.38 ± 9.56	-9.01±9.75	-10.40±9.52	0.77
Right Internal Rotation (degrees), mean ± SD	First	-4.69 ± 11.63	-2.74±11.15	-10.11±11.87	0.38
	Second	-8.03 ± 11.76	-7.71±10.34	-8.92±15.77	0.71

**Table 6:** Pre-to-postoperative changes in posture based on program adherence rate. P-values for continuous variables were calculated using Mann-Whitney U tests and are accompanied by their corresponding U-values. P ≤ 0.05. AC: acromioclavicular; EOP: external occipital protuberance; SD: standard deviation. *=significant Postural changes were measured between the first postoperative visit (two to four weeks post-op) and the second postoperative visit (eight to 12 weeks post-op), corresponding to a period of approximately six weeks between visits.

Variable	Postop Visit Number	All (n=34)	High (n=8)	Medium (n=10)	Low (n=2)	p-Value
Wall to Left Tragus (cm), mean ± SD	First	-0.87±1.84	-0.98±1.87	-0.85±1.80	-0.52±3.22	0.96
	Second	-1.84±3.30	-0.20±2.32	-2.94±3.27	-2.92±6.06	0.2
Wall to Right Tragus (cm), mean ± SD	First	-0.88±2.09	-1.28±2.14	-0.75±1.86	0.02±4.05	0.73
	Second	-1.94±3.65	-0.46±2.54	-3.23±3.91	-1.43±5.97	0.28
AC Joint (cm), mean ± SD	First	-0.47±5.29	-0.72±2.86	-0.81±6.51	2.23±8.80	0.77
	Second	-2.13±5.85	-2.35±3.69	-2.32±6.95	-0.32±10.57	0.91
Scapular Tip (cm), mean ± SD	First	-3.18±3.80	-2.69±2.96	-3.67±4.71	-2.66±2.81	0.86
	Second	-4.28±4.30	-5.40±3.77	-3.54±4.99	-3.46±3.24	0.66
EOP to Left Scapula (cm), mean ± SD	First	-2.39±4.30	-3.84±5.23	-0.86±3.41	-4.26±2.88	0.29
	Second	-4.68±4.88	-4.18±6.93	-4.69±3.32	-6.65±2.75	0.83
EOP to Right Scapula (cm), mean ± SD	First	-2.68±3.53	-3.97±3.95	-1.33±2.91	-4.24±3.74	0.24
	Second	-5.77±4.87	-4.88±5.93	-6.14±4.43	-7.50±3.79	0.77
Left Internal Rotation (degrees), mean ± SD	First	-3.92±8.19	-2.91±5.63	-4.22±10.60	-6.50±4.24	0.86
	Second	-8.92±8.91	-3.09±7.87	-12.63±8.18	-13.68±4.14	0.047*
Right Internal Rotation (degrees), mean ± SD	First	-2.30±9.20	-1.38±7.54	-3.11±11.48	-1.93±3.20	0.93
	Second	-5.91±11.45	1.14±11.50	-10.92±9.85	-9.05±4.74	0.07

**Table 7:** Pre-to-postoperative changes in posture. This table summarizes changes in postural metrics across all participants (rehab and non-rehab groups combined) at three time points: preoperative, first postoperative visit (two to four weeks post-op), and second postoperative visit (eight to 12 weeks post-op). P-values were calculated using one-way repeated-measures ANOVA (F-values). P ≤ 0.05. AC: acromioclavicular; EOP: external occipital protuberance; SD: standard deviation *=significant

Variable	Preop (n=34)	First Postop Visit (n=34)	Second Postop Visit (n=34)	p-Value
Wall to Left Tragus (cm), mean ± SD	13.54±2.68	15.02±3.30	12.57±2.69	0.23
Wall to Right Tragus (cm), mean ± SD	13.76±2.77	15.24±4.41	12.49±2.87	0.28
AC Joint (cm), mean ± SD	30.70±2.43	29.99±2.59	29.98±3.15	0.83
Scapular Tip (cm), mean ± SD	21.38±3.41	15.72±3.09	18.69±2.83	0.003*
EOP to Left Scapula (cm), mean ± SD	30.68±1.74	29.73±2.39	26.84±5.21	0.079
EOP to Right Scapula (cm), mean ± SD	30.99±1.86	29.46±1.94	27.02±4.82	0.052
Left Internal Rotation (degrees), mean ± SD	20.75±9.22	22.49±6.91	17.84±6.07	0.43
Right Internal Rotation (degrees), mean ± SD	20.34±8.25	22.78±6.73	18.96±9.96	0.62

**Table 8:** Exercise Rehabilitation Program Adherence by Living Situation Among Participants in the Rehab Group This table includes only participants in the rehabilitation group (n=20) and compares exercise adherence by living situation. P-values were calculated using Pearson’s chi-square tests (χ²) and are presented alongside their respective test statistic values. P≤0.05.

Variable	All (n=20)	With Parents (n=14)	Independent (n=6)	p-Value
High	8 (40)	4 (28.57)	4 (66.67)	0.11
Medium	10 (50)	8 (57.14)	2 (33.33)	0.33
Low	2 (10)	2 (14.29)	0 (0)	0.33

## Discussion

The aim of this pilot study was to evaluate the feasibility and initial effectiveness of a postoperative exercise rehabilitation program for adult transmasculine patients undergoing chest masculinization surgery. We found that the high adherence rates to the rehabilitation program and the participants' enthusiasm for the program - indicated by a high desire to continue the program - highlights its practicality and acceptability. These findings suggest that such a program can be successfully integrated into postoperative care for this patient population.

Our main findings highlighted significant improvements in scapular tip distance. The decrease in scapular tip distance emphasized a more posterior scapular position, although this metric alone does not establish dynamic scapular retraction which involves a complex scapulothoracic rhythm and patterns of muscle activation. Additionally, we observed improvements in wall-to-tragus distance and EOP to scapular tip distance, although these improvements did not reach statistical significance in our sample population. These three measures typically occur in tandem due to upper back, neck and head mechanics with postural changes. The trend in improvement underscores the potential benefits of chest masculinization surgery combined with the rehabilitation program, although further data is needed to better elucidate the relationship. Stratifying by level of program adherence, hand dominance, or weight of resected breast tissue did not result in further metrics showing statistically significant differences. 

Several factors may account for these findings, including the smaller sample size, duration of the study, and the specific exercises included in the rehabilitation program. As this was a pilot study, our primary focus was on the feasibility of the program. As such, a larger sample size, exercise modifications, and a longer follow-up period may be necessary to detect more statistically significant differences in posture. 

Of course, it is essential to recognize that posture correction through exercise is a nuanced and often elusive goal. Postural habits are deeply ingrained and are influenced by a myriad of factors, including neurology, biomechanics, and even psychological elements [8-13]. Studies suggest that posture is less about static positions and more about dynamic movement patterns, which makes 'correction' through isolated exercises challenging [8-13]. While some improvements can be achieved, the effectiveness of corrective exercises in permanently altering posture remains limited.

Additionally, the assumption that poor posture is a primary cause of pain has been increasingly questioned, with evidence showing that posture is only one of many factors that contribute to discomfort and dysfunction [9,14-16]. Therefore, while our study shows trends toward improvement, these findings should be interpreted with caution, as the broader literature suggests that posture correction may not always be feasible or necessary for pain relief or functional improvement.

High adherence rates and participants' reported desire to continue the exercise program indicate a positive reception and suggest potential benefits not captured by quantitative measures. Qualitative feedback from participants highlighted their positive experiences and perceived benefits of the rehabilitation program. Although this study did not measure other potential benefits of exercise rehabilitation, existing literature on postoperative exercise interventions in other contexts, such as breast cancer surgery, demonstrates significant psychosocial benefits. For example, research has shown that exercise interventions can lead to improvements in anxiety, depression, body image, mental well-being and stress among women undergoing breast cancer surgery [17-20].

Similarly, studies on gender-affirming surgeries have found that such interventions are associated with better mental health outcomes, including reduced psychological distress, lower rates of tobacco smoking, and decreased suicidal ideation [21,22]. These findings suggest that incorporating exercise rehabilitation programs in the postoperative care of transmasculine patients undergoing chest masculinization surgery could offer similar psychosocial benefits, enhancing both physical and mental well-being. Future research should explore these additional benefits and incorporate comprehensive postoperative outcome measures to provide a holistic view of the program's impact. 

Existing literature on postoperative exercise rehabilitation in other contexts also supports the importance of rehabilitation in improving functional outcomes and quality of life [19-21]. Other studies have shown various benefits of pre- and postoperative exercise programs for various surgeries including use of fewer postoperative rehabilitation services, short-term improvements in bodily pain domains of health-related quality of life and overall physical function highlighting the potential benefits of structured exercise programs [20,23,24]. Drawing parallels between these findings and the potential benefits for transmasculine individuals undergoing chest masculinization surgery highlights the relevance of such programs for this patient population. Implementing exercise rehabilitation programs for transmasculine patients can provide comprehensive benefits, enhancing both physical recovery and mental health. 

Future directions

Streamlining the process for implementation of this exercise rehabilitation program for future patients is essential despite the high adherence rates achieved. Our program involved having one healthcare provider deliver patient education on the benefits of exercise and instructions for the rehabilitation program through printed materials, videos, and in-person counseling at each follow up appointment. In the future, we aim to have comprehensive training programs for all healthcare providers involved in gender-affirming surgery postoperative management, including physical therapists, to appropriately prescribe, modify and monitor the exercises. 

Utilizing telehealth platforms could also facilitate virtual rehabilitation sessions and real-time monitoring. This would allow practitioners to better assess if patients are performing the exercises correctly and at the appropriate frequency instead of only using a standard subjective questionnaire. We also aim to collect and analyze patient feedback to enable continuous improvement of the program, ensuring it meets patient needs and optimizes outcomes.

Lastly, the importance of using validated instruments for measuring postural changes and other outcomes cannot be overstated. Long-term follow-up studies are recommended to assess the sustainability of the observed improvements and the long-term benefits of postoperative exercise rehabilitation. By addressing these areas, future research can build on the findings of this pilot study and contribute to the optimization of care for transmasculine patients undergoing chest masculinization surgery. 

It is crucial to continue this line of research with a more extensive study to validate these preliminary findings. This would provide a more comprehensive understanding of the program's effectiveness. Future research should explore these additional benefits and incorporate comprehensive postoperative outcome measures to provide a holistic view of the program's impact.

Limitations

This study has several limitations to acknowledge. First, the sample size was relatively small, which may limit the generalizability of our findings. The study's short duration and limited follow-up period may have influenced statistical significance, or lack thereof, for differences in postural metrics between study and control groups. Additionally, the specific exercises included in the rehabilitation program may not have been sufficient to elicit significant changes in all postural metrics. 

The reliance on patient self-reports for adherence and satisfaction introduces potential bias, and future studies should incorporate objective measures of adherence. Additionally, patient-reported satisfaction in this pilot may reflect acceptability and nonspecific expectancy effects of exercise rather than true posture change, and our questionnaire was not a validated instrument for perceived postural improvement. As participants self-selected into the program and there was no randomized controls, such as generic aerobic exercise, we cannot attribute subjective improvements to the specific rehabilitation protocol. Moreover, self-selection bias, the willingness to participate in the exercise program, and variations in physical activity levels outside of the prescribed exercises may serve as confounding factors. Finally, the absence of measurements for other potential benefits of exercise rehabilitation, such as psychosocial improvements, limits the comprehensiveness of our findings. 

## Conclusions

Our pilot study demonstrates the feasibility and initial effectiveness of a postoperative exercise rehabilitation program for transmasculine patients undergoing chest masculinization surgery. It is the first study to measure postural changes and feasibility related to exercise in the transgender population. The findings should primarily be used for hypothesis generation and are contingent on confirmation with validated outcome tools. Our pilot study showed high adherence rates and positive participant feedback, which indicate that such a program is both practical and acceptable. While significant improvements were observed in certain postural metrics, the overall lack of statistically significant differences underscores the need for larger sample sizes, longer follow-up periods, and potential modifications to the exercise program in future research.

Streamlining the implementation of this rehabilitation program through standardized protocols, comprehensive provider training, and the use of telehealth platforms can enhance its accessibility and effectiveness. Future studies should also explore the psychosocial benefits of postoperative exercise rehabilitation to provide a holistic view of its impact. By addressing these areas, future research can build on these preliminary findings and contribute to the optimization of care for transgender and gender-nonconforming patients undergoing chest masculinization surgery, ultimately improving patient outcomes and quality of life.
